# Linking habitat suitability to demography in a pond-breeding amphibian

**DOI:** 10.1186/s12983-015-0103-3

**Published:** 2015-05-14

**Authors:** Bianca Unglaub, Sebastian Steinfartz, Axel Drechsler, Benedikt R Schmidt

**Affiliations:** Zoological Institute, Department of Evolutionary Biology, Unit Molecular Ecology, Technische Universität Braunschweig, Mendelssohnstraße 4, Braunschweig, 38106 Germany; Department of Animal Ecology and Conservation, Biocentre Grindel, University of Hamburg, Martin-Luther-King Platz 3, Hamburg, 20146 Germany; Department of Animal Behaviour, Bielefeld University, Morgenbreede 45, Bielefeld, 33619 Germany; Institute of Evolutionary Biology and Environmental Studies, University of Zurich, Winterthurerstrasse 190, Zurich, 8057 Switzerland; KARCH, Passage Maximilien-de-Meuron 6, Neuchâtel, 2000 Switzerland

**Keywords:** Environmental niche model, Habitat suitability index (HSI), Species distribution, Reproduction probability, Survival probability, *Triturus cristatus*

## Abstract

**Introduction:**

Elucidating the relationship between habitat characteristics and population parameters is critical for effective conservation. Habitat suitability index (HSI) models are often used in wildlife management and conservation practice assuming that they predict species occurrence, abundance and demography. However, the relationship between vital rates such as survival and reproduction and habitat suitability has rarely been evaluated. In this study, we used pond occupancy and mark-recapture data to test whether HSI predicts occupancy, reproduction and survival probabilities. Our model species is the great crested newt (*Triturus cristatus*), a pond-breeding amphibian protected under the European Habitats Directive.

**Results:**

Our results show a positive relationship between the HSI and reproduction probability, whereas pond occupancy and survival probabilities were not related to HSI. Mortality was found to be higher during breeding seasons when newts are in ponds than during terrestrial phases of adult newts.

**Conclusion:**

Habitat suitability models are increasingly applied to wildlife management and conservation practice. We found that the HSI model predicted reproduction probability, rather than occurrence or survival. If HSI models indicate breeding populations rather than mere species occurrences, they may be used to identify habitats of higher priority for conservation. Future HSI models might be improved through modelling breeding populations vs. non-breeding populations rather than presence/absence data. However, according to our results the most suitable habitat is not necessarily the habitat where demographic performance is best. We recommend that conservation practitioners should use HSI models cautiously because there may be no direct link between habitat suitability, demography and consequently, population viability.

**Electronic supplementary material:**

The online version of this article (doi:10.1186/s12983-015-0103-3) contains supplementary material, which is available to authorized users.

## Introduction

Understanding the relationship between habitat quality and demography is central to the monitoring, management and recovery of threatened species. Species distribution models, also known as ecological niche models or habitat suitability models, relate species occurrence data to environmental variables. These models provide useful information on the ecological requirements of species and are widely used to predict species distribution, making them valuable tools for habitat management, impact assessment and conservation practice [1-3].

For practical application and habitat suitability assessments in the field, the output of statistical species distribution models has often been simplified to habitat suitability indices (HSI). These indices are based on habitat characteristics that can easily be measured in the field or derived from digital maps [4]. A HSI is a numerical index, ranging from 0 (unsuitable habitat) to 1 (optimal habitat). In the application of HSI models for management purposes, it is often assumed that habitat suitability predicts species performance and demography [5]. However, the most suitable habitat or habitats where density is high do not necessarily constitute habitats where demographic performance is best [6,7]. Moreover, despite being important for the management of threatened species, whether habitat suitability is associated with species occurrence [8-12] and demographic parameters, e.g. reproductive success [5,13,14] and apparent survival [15,16], has rarely been evaluated. Such tests are important because several studies did not find the expected link between habitat suitability and species occurrence or demography [9,11,17]. Species may not occur in suitable patches when structured as a metapopulation [18] or they may be found in unsuitable patches (i.e. so called sinks, [19]). To predict how species may respond to variation in habitat quality, it is necessary to understand the demographic processes through which the environment influences distributions and population dynamics [19-21]. Hence, vital rates can potentially be informative when validating HSI models [22].

In this study, we contribute to the validation of simple and easily applicable HSI models as predictive tools for management purposes. We studied the relationship between a commonly used HSI [23] and occurrence/demographic parameters in the great crested newt (*Triturus cristatus*). While a positive relationship between the HSI and newt abundance was reported [23], this assessment of the HSI is problematic as abundance is not necessarily a good indicator of habitat quality and abundance indices ignore imperfect detection [6,24]. Evidence for a relationship between HSI and newt abundance or newt occurrence is mixed. While there was no relationship between abundance indices and HSI in [25], a study on great crested newt pond occupancy that accounted for imperfect detection found that HSI predicted newt occurrence [12]. However, HSI values differed only slightly between ponds with and without newts (mean HSI ± SD: 0.70 ± 0.12 and 0.61 ± 0.13, respectively [12]). Here, we used three variables that are often used to describe the state of animal populations: species occurrence, occurrence of reproduction and survival. At the phenomenological level, we tested whether the simple HSI for great crested newts, which is based on only ten habitat characteristics, predicts species occurrence even though many habitat characteristics are known to influence occurrence of crested newt populations (e.g. [26-28]). Since a species may be found in low quality habitats within a metapopulation (i.e. sinks [7]), we further tested the predictive value of the HSI at a mechanistic level by assessing the relationship between HSI and occurrence of reproduction as well as between HSI and apparent survival.

We selected a HSI for an amphibian species because amphibians are the most endangered vertebrates [29] requiring both terrestrial and aquatic habitats during their life cycles, thus making them a particularly well suited indicator group for habitat quality [30]. Great crested newts are protected under the European Habitats Directive and may serve as umbrella species for wetland conservation [28]. If the HSI can identify habitats where demographic performance is good, it could be used to select habitats harbouring healthy populations (e.g. so called source populations [7]) for conservation purposes. However, if the HSI and demography are unrelated, then this would call for refined habitat suitability models.

## Results

We recorded capture histories of 1838 individuals from 2009 to 2011 in our study area, of which 124 individuals were recaptured at least once. Adult newts were captured at 18 sites, ranging from one to 507 individuals per pond. Larvae were found at 13 sites, ranging from one to 105 individuals caught on a single capture event. At six sites we found merely adult newts without larvae whereas only larvae were detected at one pond. At three sites we detected neither adults nor larvae. HSI values ranged from 0.43 to 0.93 in 2009, from 0.41 to 0.93 in 2010 and from 0.44 to 0.94 in 2011 for surveyed sites (Table 1).

**Table 1:** **Number of captured newts (** ***Triturus cristatus*** **) and HSI values for 22 sampling sites surveyed between 2009 and 2011**

**Sampling site**	**No. of adult newts**	**Max no. of larvae**	**HSI 2009**	**HSI 2010**	**HSI 2011**
1	118	0	0.46	0.46	0.46
2	7	0	0.45	0.41	0.46
4	1	0	0.61	0.61	0.61
8	1	0	0.61	0.61	0.61
9	45	1	0.83	0.82	0.83
10	145	4	0.93	0.93	0.93
11	325	5	0.91	0.93	0.94
12	19	0	0.56	0.56	0.56
13	0	13	0.75	0.76	0.68
13b	104	105	0.93	0.84	0.84
14	191	49	0.79	0.80	0.80
15	27	3	0.83	0.83	0.80
16	0	0	0.47	0.48	0.48
17	52	3	0.78	0.80	0.79
18	122	3	0.76	0.78	0.78
19	0	0	0.43	0.43	0.44
20	79	6	0.82	0.82	0.79
21	57	1	0.66	0.74	0.76
A	8	2	0.50	0.52	0.51
B	32	0	0.53	0.53	0.54
C	507	18	0.55	0.54	0.54
D	0	0	0.48	0.48	0.48

Sampling sites, number of captured adult newts, maximum number of larvae caught on a single capture event and HSI values for 3 years of CMR study (2009–2011).

### Modelling occupancy and reproduction probabilities

We first selected a model that best explained detection probability, while keeping occupancy and reproduction probabilities constant. Akaike model weights (*w*) suggested that model {*ψ* (.), *R* (.), *δ*_s_, *p*^[1]^ (CE), *p*^[2]^ (CE)} was best supported by the data (*w* = 0.93; see Additional file 1), whereas remaining models received little support (*w* ≤ 0.04). The number of capture events (CE) was included in the top ranking model, indicating that sampling effort positively influences the probability to detect newts in waters occupied without reproduction (logit (*p*^[1]^) =-1.59 (SE = 1.06) + 0.40 (SE = 0.19) x CE) as well as in waters with successful reproduction (logit (*p*^[2]^) = 0.72 (SE = 0.63) + 0.38 (SE = 0.20) x CE). The probability of detecting newts was generally higher for a site with reproduction (0.75 - 0.98 for CE = 1–8) than without reproduction (0.23 - 0.83 for CE = 1–8). The probability of correctly identifying sites as breeding sites increased gradually from the start to the end of breeding seasons (*δ* = 0.10 (SE = 0.07), *δ* = 0.56 (SE = 0.14), *δ* = 0.80 (SE = 0.13) and *δ* = 0.89 (SE = 0.07) for early May, late May, early June and late June, respectively).

**Figure 1:**
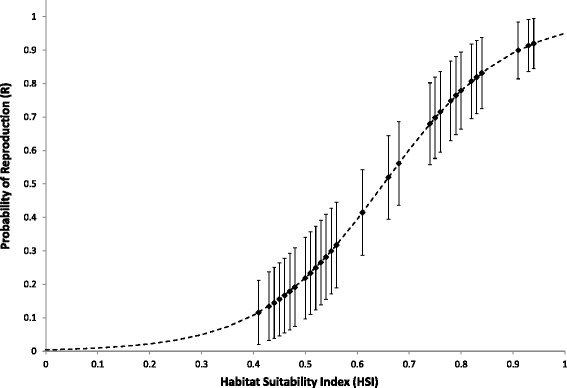
Relationship between HSI and reproduction probability of great crested newts. Symbols represent estimates and SE. Reproduction probabilities were estimated for HSI values observed at 22 ponds over 3 years.

In the second step of the analysis, we used the structure of the top-ranking model for detection probabilities and determined the effect of the HSI on *ψ* and *R*. Model {*ψ* (HSI), *R* (HSI), *δ*_s_, *p*^[1]^ (CE), *p*^[2]^ (CE)} best explained the data (*w* = 0.77; Table 2). However, while the effect of the HSI on reproduction probability was well supported by the data, the confidence interval of the estimate of the positive effect of the HSI on occupancy probability included zero (Table 3). The probability of reproduction was higher in ponds with higher HSI values (Figure 1).

**Table 2:** **Selection of multiseason-multistate models for estimating occupancy and reproduction probabilities of great crested newts**

**Model**	**AIC**	**∆AIC**	***w***	***K***
ψ (HSI), R (HSI), δ_s_, p ^[1]^ (CE), p ^[2]^ (CE)	261.34	0.00	0.77	12
ψ (.), R (HSI), δ_s_, p ^[1]^ (CE), p ^[2]^ (CE)	263.89	2.55	0.22	11
ψ (HSI), R (.), δ_s_, p ^[1]^ (CE), p ^[2]^ (CE)	269.31	7.97	0.02	11
ψ (.), R (.), δ_s_, p ^[1]^ (CE), p ^[2]^ (CE)	278.04	16.70	0.00	10

Probability of pond occupancy (*ψ*) and probability of reproduction, given presence (*R*) were held constant (.) or modelled as functions of habitat suitability index (HSI). The structure of the top-tanking model for detection probabilities {ψ (.), R (.), δ_s_, p ^[1]^ (CE), p ^[2]^ (CE)} was used to evaluate the effect of HSI on *ψ* and *R*. Probability of correctly identifying a site as breeding site, given successful reproduction (*δ*) was modelled different in each capture period and probabilities of detecting occupancy, given occupancy without reproduction (*p* ^[1]^) and with successful reproduction (*p* ^[2]^) were modelled as functions of the number of capture events per capture period (CE). AIC: Akaike’s information criterion; ∆AIC: difference of the AIC value of the current and the best model; *w*: AIC weight; *K*: number of parameters.

**Table 3:** **Parameter estimates (on the logit scale) of the top ranking multiseason-multistate model for estimating occupancy and reproduction of great crested newts**

**Logit link function**	**Beta**	**Estimate**	**95% CI**
logit (ψ_i_) = β_INT_ + β_HSI_ x HSI	β_INT_	-4.04	-9.27 – 1.20
	β_HSI_	9.38	-0.36 – 19.12
logit (R_i_) = β_INT_ + β_HSI_ x HSI	β_INT_	-5.50	-9.70 – 1.29
	β_HSI_	8.44	2.24 – 14.65

*ψ*: probability of pond occupancy; *R*: probability of reproduction, given presence. INT: Intercept; HSI: Habitat Suitability Index. CI: confidence interval.

### Modelling survival probabilities

The general model with time-dependent apparent survival and recapture probabilities (*Φ*_t_, *p*_t_) fitted the data well (goodness-of-fit test results: *χ*^2^ = 7.74, *DF* = 44, *P* = 1). There was neither evidence for transients (GOF test: *z* = 0.36, *P* = 0.72) nor an effect of capture at a previous occasion (GOF test: *z* =-0.48, *P* = 0.63).

Akaike model weights (*w*) suggested that the model assuming a seasonal effect on apparent survival {*Φ*_s_, *p* (.)} was best supported by the data (*w* = 0.987; Table 4). Monthly survival probabilities were lower during the months that newts spend in the pond (aquatic phase; March – June: *Φ*_aqu_ = 0.54; 95% CI = 0.44 – 0.63) than the months that adults spend in their terrestrial habitat (terrestrial phase; July – February: *Φ*_terr_ = 0.99; 95% CI = 0.42 – 1.00; Table 5). A model with an effect of HSI on survival probabilities was not well supported by the data (∆AICc ≥ 8.79). Annual apparent survival was calculated using the formula: *Φ*_aqu_^4^ * *Φ*_terr_^8^ [31]. The corresponding standard error was calculated by applying the delta method [32]. Annual survival probability was 0.08 ± 0.0006.

**Table 4:** **Selection of Cormack-Jolly-Seber models for estimating apparent monthly survival of great crested newts**

**Model**	**AICc**	**∆AICc**	***w***	***K***
Φ_s_, p (.)	1316.17	0.00	0.987	3
Φ_s_ (HSI), p (.)	1324.95	8.79	0.012	3
Φ_y_, p (.)	1331.27	15.11	0.001	4
Φ_y_ (HSI), p (.)	1335.81	19.65	0.000	5
Φ (HSI), p (.)	1344.33	28.16	0.000	3
Φ (.), p (.)	1345.94	29.77	0.000	2

Survival probability (*Φ*) was modelled as constant (.), as varying between years (y) or between seasons (s), i.e. during months of terrestrial and aquatic phases of adult newts. In each of this scenarios *Φ* was also modelled as function of habitat suitability index (HSI). Capture probability (*p*) was modelled as constant (.). AICc: corrected Akaike’s information criterion; ∆AICc: difference of the AICc value of the current and the best model; *w*: AICc weight; *K*: number of parameters.

**Table 5:** **Parameter estimates of the top-ranking Cormack-Jolly-Seber model for estimating survival probabilities of great crested newts**

**Model parameter**	**Estimate**	**95% CI**
Φ_aqu_	0.54	0.44 – 0.63
Φ_terr_	0.99	0.42 – 1.00
p (.)	0.07	0.05 – 0.10

*Φ*_aqu_: monthly survival probability during aquatic phases of adult newts (March – June); *Φ*_terr_: monthly survival probability during terrestrial phases of adult newts (July – February); *p*: capture probability. HSI: Habitat Suitability Index. 95% CI: 95% confidence interval.

Our estimates of apparent survival were probably distorted by emigration. Apparent survival is the product of true survival and (1 – probability of emigration) [33]. Thus, the annual probability of emigration can be calculated as 1 – (apparent survival / true survival). Based on the annual survival estimate for a metapopulation of crested newts in a similar capture-mark-recapture study [34], we assumed that true survival would be around 0.55. The annual probability of emigration would then be 0.85. If we use annual survival rates 10% lower (0.45) or higher (0.65) than the published estimate [34], then the estimates of the probability of emigration would be 0.82 and 0.87, respectively.

## Discussion

### HSI and population parameters in great crested newts (*Triturus cristatus*)

Our results suggest that the HSI for great crested newts is not related to survival or pond occupancy probabilities, but that newt populations are more likely to breed in ponds with higher HSI values. In contrast, [23] showed that there is a positive relationship between the HSI and an index of newt abundance. These results could be misleading, however, for two reasons. First, abundance can be a misleading indicator for habitat quality [6]. Second, imperfect detection was not taken into account [24]. For example, [23] acknowledged that macrophyte cover in ponds may have biased their results. The positive relationship between HSI and abundance reported in [23] could not be confirmed by [25]. In our study, we therefore selected different variables to describe the state of newt populations and we accounted for imperfect detection.

We found that the best multiseason-multistate occupancy model included HSI as covariate for both the probabilities of occupancy (*ψ*) and of reproduction (*R*). A previous study which did not differentiate between the presence/absence of adults and larvae [12] found that HSI predicted pond occupancy. In our study, the top-ranking model also included a positive effect of HSI on pond occupancy but the confidence interval of this estimate included zero (Table 3). The comparison of our results with those of [12] shows that differentiating between presence/absence of adults and presence/absence of larvae can give additional insights into habitat suitability. Our results suggest that the HSI is a good predictor for reproduction but not for pond occupancy.

We also found newts in ponds with very low HSI values, seemingly not representing suitable habitat (Table 1). Bentonite mats, applied to the soil of one pond in 2001, prevented periodical drying and allowed for the existence of large fish populations. Occasional high water levels led to the colonization of formerly fishless ponds by native and invasive fish. Fish are well known to negatively affect the distribution and abundance of great crested newts and other amphibians (see reviews in [28] and [35]) which is why the presence of fish leads to lower HSI values for affected ponds (see SI_7_). Accordingly, the number of captured newts decreased in those waters over our monitoring period, but still we regularly found some adult newts even in ponds with predatory fish. However, ponds occupied by predatory fish are unlikely to represent suitable habitat for crested newts. Since the goal of HSI models is to predict suitable habitat rather than mere species occurrence, they should not simply indicate whether a pond is occupied or not.

The model developed by [23] emphasizes primarily the aquatic habitat where adult newts congregate for a few months during spring and early summer to reproduce [36]. As would therefore be assumed, our results suggest that the HSI represents a good tool to detect ponds where newts are more likely to reproduce successfully (Figure 1). Hence, it is probable that newts occurring in ponds with lower HSI values do not breed, or that larvae do not survive in these waters until metamorphosis. As spatial variation in newt reproductive success may be common [37], the HSI could allow conservation managers to identify breeding populations or, conversely, populations constituting sinks because of a lack of reproduction [7]. This kind of information is certainly valuable for the effective conservation and recovery of threatened species. Taken together, our results for occupancy and reproduction probabilities suggest that the HSI does a good job because it appears to differentiate between populations with high and low probabilities of reproduction.

If the HSI indicates suitable habitat, then one may expect a positive relationship between HSI and survival. However, we did not find such a relationship (Table 4). Ponds with higher HSI values appear to hold larger populations [23] and survival might be negatively affected by density dependence. Other environmental factors, such as climate may have a stronger effect on survival than habitat quality [34]. To date, there is no data that would allow to test this, or any other hypothesis, for the absence of an effect of the HSI on survival. Remarkably, annual apparent survival was low in our study, suggesting that about 85% of great crested newts may have emigrated. This is an extraordinarily large proportion. However, emigration in the context of our study refers to the place where the newts were captured (i.e. the ponds) and is therefore emigration from the breeding population rather than emigration from the study area. In other words, if a newt did not enter the pond anymore during the three years of our study, it was considered an emigrant. In a short-term study such as ours, it is not possible to distinguish between temporary and permanent emigration [38,39]. Newts may have avoided the ponds and skipped reproduction in the later years of our study in response to the invasion and increase of fish populations. This interpretation of the emigration rate as skipping reproduction in some years is supported by the fact that we observed very few cases of among-pond movement (i.e. 11 individuals). Skipping reproduction might be a strategy to deal with predatory fish because survival on land was high and ponds were temporary, and therefore fish-free, in the past. To test this hypothesis, it would be necessary to extend the mark-recapture study to the terrestrial habitat. To our knowledge, the present study is the first to directly compare survival probabilities during aquatic and terrestrial phases of pond-breeding amphibians. Our study shows that survival probabilities were lower during aquatic than during terrestrial phases of adult great crested newts (monthly survival 54% and 99%, respectively). Monthly survival of 99% (or 92% across the eight months of the terrestrial phase) is unexpectedly high but [40] reported an estimate of annual survival of 99.6% in a cohort of *Ambystoma maculatum* salamanders. While our results suggest that mortality occurs primarily in the water during the breeding season, [36] found that annual survival in a metapopulation of crested newts in the UK was determined by winter weather, i.e. environmental conditions during the terrestrial phase of adults. Hence, factors determining survival may vary spatially [41]. If spatial variation in survival is common, then conservation management should take population-specific differences into account [42].

### HSI as a general conservation tool

Habitat suitability models are increasingly applied to wildlife management and conservation planning [3,8]. Guisan et al. [3] outlined the steps that are necessary to increase the use of such models to guide conservation decisions. They noted that modelled occurrence probabilities do not always correlate with demographic processes determining population viability [21,43], a finding we regard to be of particular importance for the use of HSI models in conservation practice. We suggest that a focus on the occurrence of a species may not always provide the best models for conservation applications. First, a species may occur in sink habitats [7] and second, a species may not occur in suitable patches as a consequence of extinction and colonization dynamics in metapopulations [18] or due to interspecific competition [19]. Consequently, it is not surprising that many studies did not find the expected correlation between modelled habitat suitability and individual performance, demography or population viability ([9, 11, 17 and references in [3]). Establishing a link between reproduction and habitat suitability seems to be an important step forward [14]. We suggest that modelling the probability of reproduction rather than the probability of occurrence in habitat suitability models using techniques that estimate a true probability rather than a relative suitability score might give valuable additional insights [44-47]. This might be a better way to identify suitable habitat and to increase the utility of these models for conservation.

## Conclusions

HSI models are increasingly applied as predictive tools for management purposes, assuming that habitat suitability predicts species performance and demography. However, the validity of this assumption has rarely been evaluated. We studied the relationship between a commonly used HSI [23] and occurrence/demographic parameters in a pond-breeding amphibian protected under the European Habitats Directive, the great crested newt (*Triturus cristatus*). Our results show a positive relationship between the HSI and reproduction probability (i.e. the occurrence of larvae), whereas pond occupancy and survival probabilities were not related to HSI. This is both good and bad news for conservation managers. The good news is that HSI models may indicate breeding populations rather than mere species occurrences, thus identifying habitats of higher priority for conservation purposes. Modelling breeding populations vs. non-breeding populations rather than presence/absence data might help to identify habitats harbouring healthy populations and to improve the utility of HSI models for the conservation of threatened species (for similar conclusions, see [48,49]). The bad news is that the most suitable habitat is not necessarily the habitat where demographic performance is best. Since there may be no direct link between habitat suitability and demographic processes determining population viability, we recommend that conservation practitioners should use HSI models cautiously.

## Methods

### Study species and determination of HSI

The great crested newt (*Triturus cristatus*) is a pond-breeding amphibian species, listed in Annexes II and IV of the European Habitats Directive (92/43/EEC). EU member states are therefore required to monitor the conservation status of this species. Accordingly, monitoring and management of great crested newt populations would benefit from informative and easily applicable tools and consequently, from a validated HSI. The HSI for the great crested newt incorporates ten habitat features (see Additional file 2; [23]), which are assessed for a pond and converted to suitability index (SI) scores on a scale from 0.01 to 1.0. SIs are site location relative to species distribution (i.e. whether a population occurs at the edge or in the centre compared to the distributional range; SI_1_), pond area (SI_2_), pond permanence (SI_3_), water quality (SI_4_), shading of pond perimeter (SI_5_), number of water fowl per 1000 m^2^ (SI_6_), impact of fish (SI_7_), pond density within a radius of 1 km (SI_8_), proportion of suitable terrestrial habitat within surrounding 500 m (SI_9_) and macrophyte cover (SI_10_). The HSI for great crested newts is calculated as geometric mean of these ten suitability indices:

$$ \mathrm{H}\mathrm{S}\mathrm{I} = {\left(\mathrm{S}{\mathrm{I}}_1\ast \mathrm{S}{\mathrm{I}}_2\ast \mathrm{S}{\mathrm{I}}_3\ast \mathrm{S}{\mathrm{I}}_4\ast \mathrm{S}{\mathrm{I}}_5\ast \mathrm{S}{\mathrm{I}}_6\ast \mathrm{S}{\mathrm{I}}_7\ast \mathrm{S}{\mathrm{I}}_8\ast \mathrm{S}{\mathrm{I}}_9\ast \mathrm{S}{\mathrm{I}}_{10}\right)}^{1/10} $$

We calculated the HSI for each pond in each year of the study. Since this index was originally developed for the UK, we had to transfer the statements regarding the location relative to species distribution (SI_1_) to Germany. According to [23], study sites with a high probability of great crested newt occurrence within each 10 km square are scored with 1.0 for SI_1_ and sites with a low probability of newt occurrence are scored with 0.5. Within Germany, our study sites are located in an area of an intermediate probability of newt occurrence. Therefore, we fixed SI_1_ to 0.75 for all ponds. Since the original HSI [23] only provides values for ponds of an area of up to 2000 m^2^, we had to omit SI_2_ for eight ponds of greater size and calculated the ninth rather than the tenth root of the product instead.

### Study area and sampling procedure

**Figure 2:**
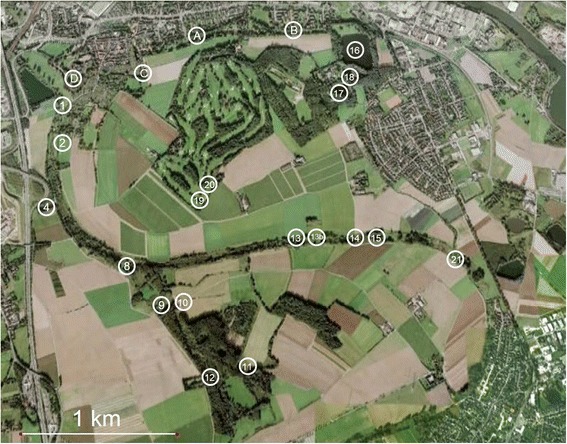
Overview of the study area near Krefeld (Germany). Illustrated are all 22 surveyed ponds which are mainly located within the FFH-area of the “Latumer Bruch” (DE-4605-301; coordinates: 51°19’5” N, 6°39’17” E).

We conducted a capture-mark-recapture (CMR) study and surveyed 22 lentic water bodies in a former flooding area of the Rhine river near Krefeld, Germany (coordinates: 51°19’5” N, 6°39’17” E; Figure 2; Additional file 3), for the presence of great crested newts. The study area is primarily dominated by grasslands, woodlands and wetlands. The northern part, however, is also influenced by adjacent residential areas and agriculture. Adult and larval crested newts can easily be captured with traps in ponds during the breeding season from March to July [50]. Detection or non-detection of adults and larvae was recorded during multiple capture events from March to June in 2009 and 2010 as well as from April to June in 2011. Several visits to each water body were essential to distinguish between sites where great crested newts did not occur and sites where this species has been overlooked [51]. Therefore, every site was visited between 12 and 66 times during the 3 years of monitoring. Newts were captured using Ortmann’s funnel traps [52], which were constructed of empty 10 liter paint buckets with four distinct openings in which half-cut inverted 1.5 liter plastic bottles were inserted functioning as funnels. These traps were evenly distributed along the shoreline and remained in the water for 48 hours. The number of traps deployed per capture event varied according to pond area, ranging from one to 36 traps. After data collection, all individuals were released immediately.

To allow individual recognition during the CMR study, we used photographs of the ventral side of newts, which provides a highly variable but individually unique and lifelong permanent colour pattern [53]. Recaptured individuals were identified automatically by the program AMPHIDENT [54]. AMPHIDENT can reliably detect recaptures even in large datasets based on a cross-correlation comparison algorithm (>1500 individuals; [55]).

### Modelling occupancy and reproduction probabilities

We used multiseason-multistate occupancy models [43,44] to estimate the probability that great crested newts occur in a water body (*ψ*) and the probability that newts reproduced successfully (*R*). The model assumes that sites fall into one of three categories: absence of the species, presence without production of offspring, and presence with production of offspring. With this model, we can model both the presence/absence of newts and the presence/absence of reproduction (given occurrence). We used the presence/absence of larvae as a proxy for production of offspring (i.e. the probability that newts reproduced successfully; larvae are more likely to indicate successful recruitment than the presence of eggs). Unlike models based on presence-only data, which estimate a habitat suitability score, these models estimate true probabilities [46]. Sampling effort varied between sites, ranging from three to 45 capture events during the entire breeding seasons. Therefore, repeated detection/non-detection data were simplified for analysis as follows. For each breeding season per year (i.e. primary period) we defined four capture periods (two-week periods: 1–15 May, 16–31 May, 1–15 June and 16–30 June). If a site was not visited during a capture period, this was treated as a missing observation. If multiple capture events were conducted within a capture period, a site was classified as state *m* = 0 if no newts were detected, as state *m* = 1 if at least one adult was found and as state *m* = 2 if at least one larva was detected. Accordingly, every site received a specific detection history like 1022 0112 0122, where 1 indicates that adults were detected, 0 indicates that neither adults nor larvae were found and 2 indicates that larvae were detected. In this way, we retained as much data as possible without having too many missing observations within a given sampling season [56]. Because of this simplification, we used capture effort (CE = number of capture events per capture period) as explanatory variable for detection probability.

We used a two-step approach to model selection. We first modelled the detection process and then the probabilities of site occupancy and reproduction. We developed an *a priori* candidate model set (see Additional file 1) to select a best model for the probability of detecting occupancy given that a site was occupied without reproduction (*p*^[1]^), the probability of detecting occupancy given that a site was occupied with successful reproduction (*p*^[2]^) and the probability of correctly identifying a site as breeding site given that successful reproduction did occur (*δ*). To identify the best detection model we held occupancy parameters (*ψ* and *R*) constant and evaluated the effect of capture effort on *p*^[1]^ and *p*^[2]^, allowing *δ* to vary in time. We hypothesized that *p*^[1]^ and *p*^[2]^ are influenced by capture effort (CE), because a higher sampling effort should result in a higher probability of detecting species occurrence. This variable accounts for the fact that the number of capture events varied both within and between ponds. Moreover, we allowed *δ* to vary between May and June (*δ*_m_) as well as between capture periods (*δ*_s_), since larvae should be more abundant and bigger later in the season and are therefore easier to detect.

In the second step, we determined the effect of the HSI on occupancy probability (*ψ*) and reproduction probability (*R*), while using the best model for the detection parameters as determined in the first step. Since we were mainly interested in the influence of the HSI on occupancy and reproduction probabilities rather than in state transitions between years, we modelled variables describing changes over time (parameters *ψ*^m^_t+1_ and *R*^m^_t+1_ in the transition probability matrices [45], with m = state) in the same way as the initial variables (*ψ*_t=1_ and *R*_t=1_). Overall, four different models were considered: a) both *ψ* and *R* were modelled as constant; b) both *ψ* and *R* were modelled as functions of the HSI; c) *ψ* was modelled as constant and *R* was modelled as a function of the HSI; d) *ψ* was modelled as a function of the HSI and *R* was modelled as constant. We hypothesized that sites with a higher HSI value should have higher probabilities of occupancy and reproduction. Statistical models were implemented in program Presence 6.2 [57].

### Modelling survival probabilities

We used Cormack-Jolly-Seber models [58] to estimate monthly survival and detection probabilities (*Φ* and *p*). Capture data were pooled for the months March, April, May and June. If a water body was not visited during a month, detection probability *p* was set to 0 for this site and period. If multiple capture events were conducted within a month, only the first capture of individuals was counted. Pooling data from several consecutive capture occasions within a month generally increases precision but may induce some bias in survival estimates [59-61]. Overall, adults were captured in 18 out of 22 water bodies (Table 1). However, at two of these 18 sites only one adult was detected, both only once in April 2010. Neither individual was ever recaptured again during the entire sampling period. Therefore, we excluded these two ponds from the mark-recapture analysis and estimated survival probabilities at 16 different sites. Data were too sparse to include covariates for detection probability. Therefore, detection probability (*p*) was always held constant even though this model may not be the best description of the observation process. Survival probabilities (*Φ*) were modelled either as constant, as varying between years, or as varying between aquatic (March - June) and terrestrial (July - February) phases of adult newts (survival was assumed to be constant within both the aquatic and the terrestrial phase). In the latter case, the specification of the unequal time intervals between capture occasions allowed for the calculation of monthly survival estimates.

For each of these scenarios, we also allowed *Φ* to be a function of the covariate HSI. We hypothesized that there is a positive correlation between the HSI and survival probabilities. Models were implemented in program MARK 6.2 [62].

Since only 11 individuals were recaptured at different sites and therefore moved from one site to another, we did not attempt to estimate dispersal probability. If an individual was detected at a new site, then it was scored as having died at the first site (by assigning “-1” to the capture history) and entered as a new individual at the new site.

### Model selection and model notation

Model selection was based on Akaike’s information criterion (AIC [63]). The model with the lowest AIC (or AICc) was considered the most parsimonious model given the data. We also used Akaike weight (*w*) as a measure of relative support for each model.

Our model notation system follows the standard notation of [58] and [64] providing information about the sources of variation used to model each parameter. The term (.) indicates that a parameter was held constant (i.e. no covariates).

## Additional files

Additional file 1:
**Model selection of multiseason-multistate models for estimating detection parameters of great crested newts.**
Additional file 2:
**Ten Suitability Indices (SI) for the calculation of the HSI for great crested newts according to Oldham et al. (2000).**
Additional file 3:
**Distance matrix [km] of surveyed ponds.**
