# Supplementary material for: Linking habitat suitability to demography in a pond-breeding amphibian
Source: Front Zool. 2015 May 14;12:9. doi: 10.1186/s12983-015-0103-3 (PMC4430901; doi:10.1186/s12983-015-0103-3)
Supplement: Additional file 1: — Model selection of multiseason-multistate models for estimating detection parameters of great crested newts. [file 12983_2015_103_MOESM1_ESM.pdf]

Additional file 1: Model selection of multiseason multistate models for estimating detection parameters of great crested newts.

Model	AIC	ΔAIC	*w*	*K*
ψ (.), R (.), δ_s, $p^{[1]}$ (CE), $p^{[2]}$ (CE)	278.04	0.00	0.93	10
ψ (.), R (.), δ_m, $p^{[1]}$ (CE), $p^{[2]}$ (CE)	284.13	6.09	0.04	8
ψ (.), R (.), δ_s, $p^{[1]}$ (.), $p^{[2]}$ (.)	285.46	7.42	0.02	8
ψ (.), R (.), δ_m, $p^{[1]}$ (.), $p^{[2]}$ (.)	291.53	13.49	0.00	6
ψ (.), R (.), δ (.), $p^{[1]}$ (CE), $p^{[2]}$ (CE)	303.64	25.60	0.00	7
ψ (.), R (.), δ (.), $p^{[1]}$ (.), $p^{[2]}$ (.)	311.31	33.27	0.00	5

Probability of pond occupancy (ψ) and probability of reproduction, given presence (R) were held constant (.) to evaluate the effects of sampling effort and sampling time on detection parameters. Probability of correctly identifying a site as breeding site, given successful reproduction (δ) was modelled as constant (.) or was allowed to vary between capture periods (s) or between months (m). Probabilities of detecting occupancy, given occupancy without reproduction ($p^{[1]}$) and with successful reproduction ($p^{[2]}$) were modelled as constant (.) or as functions of the number of capture events per capture period (CE). AIC: Akaike's information criterion; ΔAIC: difference of the AIC value of the current model and of the best model; *w*: AIC weight; K: number of parameters.
