# Supplementary material for: Linking habitat suitability to demography in a pond-breeding amphibian
Source: Front Zool. 2015 May 14;12:9. doi: 10.1186/s12983-015-0103-3 (PMC4430901; doi:10.1186/s12983-015-0103-3)
Supplement: Additional file 2: — Ten Suitability Indices (SI) for the calculation of the HSI for great crested newts according to Oldham et al. (2000). [file 12983_2015_103_MOESM2_ESM.pdf]

Additional file 2: Ten Suitability Indices (SI) for the calculation of the HSI for great crested newts according to Oldham *et al.* (2000).

SI	Factor	Explanation
SI_1	Geographic location	Location of the population relative to species national distribution
SI_2	Pond area	Pond surface area (m^2)
SI_3	Pond permanence	Frequency of pond drying per decade
SI_4	Water quality	Water quality determined by using invertebrate diversity
SI_5	Pond Shading	Percentage of perimeter shaded to at least 1m from shore
SI_6	Water fowl	Number of water fowl seen per $1000m^2$
SI_7	Fish	Occurrence and species composition of fish populations
SI_8	Pond density	Number of ponds occurring within $1km^2$ of survey pond
SI_9	Proportion of “newt friendly” habitat	Amount of shelter and foraging opportunities offered by the terrestrial habitat within 500m surrounding the pond
SI_{10}	Macrophyte content	Percentage of pond surface area occupied by macrophyte cover

SI: Suitability Index.
