# Supplementary material for: Linking habitat suitability to demography in a pond-breeding amphibian
Source: Front Zool. 2015 May 14;12:9. doi: 10.1186/s12983-015-0103-3 (PMC4430901; doi:10.1186/s12983-015-0103-3)
Supplement: Additional file 3: — Distance matrix [km] of surveyed ponds. [file 12983_2015_103_MOESM3_ESM.pdf]

Additional file 3: Distance matrix [km] of surveyed ponds.

	1	2	4	8	9	10	11	12	13	13b	14	15	16	17	18	19	20	21	A	B	C	D
1	0.00																					
2	0.33	0.00																				
4	0.70	0.37	0.00																			
8	1.20	0.93	0.66	0.00																		
9	1.60	1.34	1.07	0.42	0.00																	
10	1.64	1.40	1.14	0.48	0.13	0.00																
11	2.25	2.01	1.76	1.10	0.70	0.62	0.00															
12	2.29	2.03	1.73	1.10	0.68	0.65	0.28	0.00														
13	1.84	1.72	1.62	1.07	0.92	0.79	0.86	1.11	0.00													
13b	2.05	1.95	1.87	1.32	1.15	1.03	0.98	1.25	0.25	0.00												
14	2.32	2.23	2.16	1.62	1.43	1.31	1.17	1.45	0.55	0.30	0.00											
15	2.39	2.31	2.24	1.70	1.51	1.39	1.23	1.51	0.63	0.38	0.08	0.00										
16	2.21	2.33	2.49	2.29	2.36	2.27	2.45	2.70	1.59	1.49	1.44	1.43	0.00									
17	2.04	2.12	2.24	1.99	2.04	1.94	2.10	2.35	1.24	1.15	1.12	1.12	0.34	0.00								
18	2.13	2.22	2.35	2.12	2.17	2.07	2.23	2.48	1.38	1.27	1.23	1.23	0.22	0.13	0.00							
19	1.26	1.19	1.19	0.86	0.97	0.90	1.30	1.47	0.62	0.80	1.06	1.13	1.43	1.13	1.26	0.00						
20	1.19	1.13	1.15	0.87	1.02	0.95	1.37	1.54	0.70	0.88	1.13	1.20	1.42	1.13	1.25	0.08	0.00					
21	2.97	2.90	2.83	2.27	2.04	1.92	1.61	1.89	1.21	0.96	0.67	0.59	1.69	1.45	1.51	1.72	1.78	0.00				
A	1.21	1.42	1.69	1.78	2.04	1.99	2.43	2.60	1.66	1.74	1.88	1.92	1.11	1.08	1.11	1.13	1.06	2.41	0.00			
B	1.84	2.00	2.21	2.11	2.27	2.19	2.47	2.70	1.62	1.59	1.62	1.64	0.45	0.55	0.52	1.29	1.26	2.00	0.68	0.00		
C	0.64	0.86	1.15	1.37	1.71	1.69	2.23	2.34	1.60	1.75	1.97	2.03	1.59	1.46	1.53	0.99	0.90	2.58	0.57	1.20	0.00	
D	0.33	0.66	1.03	1.48	1.87	1.89	2.48	2.55	1.98	2.17	2.41	2.48	2.09	1.97	2.04	1.37	1.29	3.04	1.02	1.69	0.51	0.00

Pairwise distance between centers of ponds in kilometres. Darker shadings indicate greater distance.
